# Impact of rational emotive therapy on anxiety associated physical symptoms among older adults

**DOI:** 10.6026/973206300212214

**Published:** 2025-07-31

**Authors:** Ramani G., Tamizharasi K.

**Affiliations:** 1 Department of Psychiatric Nursing, Kongunadu College of Nursing, Coimbatore, The Tamil Nadu DR.M.G.R Medical University, Chennai, India; 2 Department of Paediatric Nursing, Sri Gokulam College of Nursing, Salem, The Tamil Nadu DR.M.G.R Medical University, Chennai, India

**Keywords:** Rational emotive therapy, Anxiety, older adults, physical Symptoms

## Abstract

Anxiety is one of the most common mental health issues among the older adults. This pilot study assessed the effectiveness of Rational Emotive Therapy (RET) in reducing anxiety and related physical symptoms among elderly residents of selected old age homes in Coimbatore. A quasi-experimental design was adopted with 20 participants, divided equally into experimental and control groups. The experimental group received 10 weekly RET sessions, while the control group received routine care. Anxiety levels were measured using the Hamilton Anxiety Rating Scale and physical symptoms were assessed through a checklist. Pre- and post-intervention data showed a significant reduction in anxiety scores in the experimental group (p = 0.001).

## Background:

Anxiety is one of the most prevalent yet under-recognized mental health conditions affecting the elderly population. In institutional settings such as old age homes, older adults often experience heightened anxiety due to social isolation, chronic illness, loss of autonomy and bereavement. Research indicates that anxiety disorders among the elderly may be underdiagnosed, partly due to atypical presentations and overlapping physical symptoms. Dantas *et al.* emphasized the link between physical inactivity and anxiety, highlighting the unique mental health needs of aging individuals [1]. The prevalence of anxiety in this demographic has been reported to range from 10% to over 20%, especially when using assessment tools tailored for older populations, as noted by Cisneros *et al.* in a systematic review [2]. Additionally, elderly individuals often present anxiety through somatic symptoms-such as hypertension, respiratory changes and fatigue - which may lead to misdiagnosis or delayed care, as first discussed by Turnbull and further supported by Gelenberg *et al.* [3]. Effective management of anxiety in elderly populations is essential, as untreated anxiety can significantly impair functional status, increase healthcare usage and elevate the risk of comorbid depression and mortality. This concern was extensively reviewed by Andreescu *et al.* and echoed in recent directions in geriatric mental health by Reynolds *et al.* [4]. Pharmacological options, though widely used, often carry risks such as sedation, falls and drug interactions due to age-related changes in metabolism. Mangoni *et al.* stressed the pharmacokinetic and pharmacodynamic changes that complicate drug use in older adults [5]. This highlights the need for non-pharmacological interventions that are safe, structured and adaptable for older adults. de Sá-Caputo *et al.* emphasized the importance of such approaches in their editorial on aging neuroscience [6]. Rational Emotive Therapy (RET), a form of cognitive-behavioral therapy, focuses on identifying and restructuring irrational beliefs to promote emotional well-being. Given its emphasis on developing logical thinking, disputing harmful beliefs and enhancing problem-solving skills, REBT presents a promising approach for managing anxiety among elderly individuals in residential care. Therefore, it is of interest to evaluate the effectiveness of RET in reducing anxiety and its associated physical symptoms among elderly residents of selected old age homes in Coimbatore.

## Methodology:

## Research design and setting:

A quasi-experimental pre-test and post-test control group design was used. The study was conducted at Sevalaya and Anbalaya old age homes in Coimbatore.

## Participants and sampling:

Twenty elderly individuals aged 60 and above with anxiety symptoms were selected using purposive sampling. They were divided equally into experimental (n=10) and control (n=10) groups [7].

## Inclusion and Exclusion criteria:

Inclusion criteria included age ≥60, presence of anxiety (as per HAM-A) and willingness to participate. Those with mental illness, cognitive decline, or hearing loss were excluded.

## Ethical considerations:

Ethical clearance was obtained from the Institutional Ethical Committee of Sri Gokulam Hospital. Informed consent was taken and confidentiality was ensured.

## Intervention:

The experimental group received 10 weekly one-on-one REBT sessions (60 minutes each), focusing on identifying and disputing irrational beliefs using the ABCDE model. The control group received routine care.

## Data collection tools:

[1] HAM-A Scale to assess anxiety

[2] Checklist for physical symptoms

Pre- and post-tests were conducted for both groups after 10 weeks.

## Results:

Table 1 (see PDF) displays the demographic distribution of 20 elderly participants. The majority were female, aged 66-75, with no formal education and most had a history of employment. Key social factors included limited family contact and admission to old age homes due to caregiver absence or financial hardship. Table 2 (see PDF) shows a significant drop in anxiety scores for the experimental group from 30.60 to 20.10 (18.75% reduction) after RET, while the control group showed only a minimal change from 29.70 to 29.10 (1.08% reductions). This indicates the strong effectiveness of REBT in reducing anxiety levels. Table 3 (see PDF) compares physiological symptoms such as respiration rate and blood pressure. Post-intervention, the experimental group showed improvement: high BP cases reduced from 60% to 20% and normal respiratory rates increased. The control group showed no significant changes, further supporting REBT's impact on physical symptom relief alongside anxiety reduction. Figure 1 (see PDF) illustrates the same results visually, highlighting the sharp contrast between the anxiety score improvements in the REBT group compared to the control group.

## Discussion:

This pilot study examined the impact of Rational Emotive Therapy (RET) on anxiety and its associated physical symptoms among elderly residents of old age homes. The findings revealed a statistically significant reduction in anxiety in the experimental group, with a notable 18.75% decrease in post-test scores, compared to only 1.08% in the control group. Several demographic factors were found to influence anxiety levels, aligning with trends observed in global geriatric mental health literature. Age emerged as a key factor, with participants aged 66-75 years showing the highest anxiety levels. The present study's findings align with and extend the insights of Qin *et al.* (2023), who demonstrated the effectiveness of Rational Emotive Behavior Therapy (REBT) in improving alexithymia and sleep quality in older adults residing in nursing homes, though it showed limited impact on anxiety and depression in the short term [8]. This is consistent with findings by Zhang *et al.* who reported elevated anxiety in elderly individuals entering later stages of life due to losses in independence and health [9]. Andreescu and Varon further highlighted age-related neurological changes that increase emotional vulnerability [10]. Female participants reported more anxiety than males, aligning with studies showing that elderly women often experience greater distress due to longer lifespans, caregiving roles and hormonal changes [11]. Participants with no formal education had higher anxiety, likely due to limited coping strategies and lower health literacy, as reported by Hersen & Hasselt and Creighton *et al.* [12, 13]. Furthermore, the absence of a prior work history was associated with elevated anxiety, reinforcing Boerner's argument that lack of occupational engagement may reduce purpose and identity in old age [14]. A similar association was found by Kirmizioglu *et al.* who linked occupational inactivity with poorer emotional outcomes [15]. Social isolation, measured via family visitation frequency, was another strong predictor of anxiety. Elderly residents with fewer visits had significantly higher anxiety, mirroring findings by Vadla *et al.* and Castro *et al.* who reported that low social contact contributes to loneliness, depression and increased anxiety symptoms [16, 17]. Additionally, participants with longer stays in old age homes reported more distress; supporting Salzman & Sheikh suggestion that institutionalization often leads to emotional deterioration [18]. RET significantly reduced anxiety levels, consistent with many studies highlighting its effectiveness across age groups and settings. Schenk *et al.* demonstrated that RET significantly lowered anxiety and irrational beliefs in students with generalized anxiety disorder [19], while Turner *et al.* reported large anxiety reductions after RET in clinical populations [20]. In elderly cohorts specifically, Ellis advocated the use of RET as a structured method to challenge deep-seated irrational fears and beliefs [21]. Arefi *et al.* also found that REBT was as effective as reminiscence therapy and psychodrama in reducing death anxiety in elderly men [22]. This study further showed that RET not only reduced psychological anxiety but also improved physical symptoms, such as blood pressure and respiration. Similar psychophysiological improvements were noted in studies on RET's role in reducing stress-related somatic symptoms [23] and in enhancing emotion regulation in older adults [24]. Other group-based studies, such as those by Cowan & Brunero and Widodo, confirmed REBT's value in reducing collective anxiety in structured institutional settings [25, 26]. In summary, the study confirms that RET is both effective and practical for reducing anxiety and associated physical symptoms in elderly populations. It complements existing literature supporting RET's success across various contexts, from student anxiety [27] to addiction recovery [28] and even pregnancy-related anxiety [29]. Given its cognitive and emotional adaptability, REBT holds promise as a scalable, non-pharmacological mental health strategy in geriatric care.
